# A survey on pupae parasitoid species of *Dendrolimushoui* (Lajonquiere) (Lepidoptera, Lasiocampidae) in China

**DOI:** 10.3897/BDJ.11.e97878

**Published:** 2023-03-27

**Authors:** Hao yu Lin, Ci ding Lu, Zheng hao Chen, You jun Zhou, Yun Liang, Hui Chen, Guang hong Liang

**Affiliations:** 1 Fujian Agriculture and Forestry University, Fuzhou, China Fujian Agriculture and Forestry University Fuzhou China; 2 South China Agricultural University, Guangzhou, China South China Agricultural University Guangzhou China

**Keywords:** *Cryptomeriajaponica*, *Dendrolimushoui*, parasitoid, natural enemy, defoliator

## Abstract

Cryptomeriajaponicavar.sinensis Miquel in south China is currently overwhelmingly infested by a native caterpillar species, *Dendrolimushoui* (Lepidoptera), which is causing severe economic losses and ecological disasters in both planted and natural forests. Our results include report of five parasitoid species and eight parasitoid flies within *D.houi* and a dominant endoparasitoid species *Kriechbaumerelladendrolimi*, which attacks pupae of *D.houi* with a high parasitism rate. This result might be helpful to improve better identification and application in the future for potential biological control of *D.houi* in the forests of east Asia.

## Introduction

*Dendrolimushoui* (Lajonquiere) (Lepidoptera, Lasiocampidae) is a species of caterpillar that feeds on the leaves and branch tips of coniferous species, such as Cryptomeriajaponicavar.sinensis Miquel, *Pinusyunnanensis* Faranch. and *Pinuskesiya* Royle ex Gordon (Kong et al. 2007), causing large losses of branches and leaves, preventing the normal growth and development of hosts and eventually resulting in dying forests across South Asian regions including India, Burma, Sri Lanka, Indonesia and the south of China. Caterpillars have reportedly destroyed 50,000 hectares of *C.japonica* in Zhejiang Province, China, in 2013 (Zhang 2013); thousands of hectares of *Cupressusfunebris* Endl. in Sichuan Province (He et al. 2018); and 8800 hectares of *P.kesiya* west of Yunnan Province (Yang 2015), which has significantly reduced the local forest quality and negatively affected the scenic landscape, especially in some national parks and natural reserve zones. Additionally, these caterpillars often cause painful allergies amongst tourists when contacting the venomous seta densely adhering on the larval skin (Luo et al. 1998).

The commercial insecticide Sendebao (0.18% abamectin plus *Bacillusthuringiensis* at 108 spores/g) has been broadly used to control *D.houi* in large areas, which has effectively reduced the population of caterpillars and protected local forests (Zhang 2013). Unfortunately, this chemical control has also been proven to have a negative impact on populations of natural enemy species of this caterpillar and overuse of this pesticide might lead to negative effects, including chemical resistance and resurgence of the pest species in the environment (Caniço et al. 2020). Biological control has gradually attracted the attention of entomologists due to its environmentally friendly nature. These successful examples suggest that biological control using parasitoid species can be a sustainable and effective tool for the suppression of pest populations without repeated biocontrol agent release (Vargas et al. 2007, Vargas et al. 2013). In the past three decades, 22 species of parasitic wasps and parasitic flies within *D.houi* have been discovered and identified (Lin et al. 2017, Liang et al. 2018). However, to date, little is known about the biology of any parasitoid species or about the morphology of adult and immature stages.

Here, we conducted a survey of the various parasitoid species that attack *D.houi* and further collected and identified all natural enemies of *D.houi* after laboratory rearing. The biological characteristics of parasitic species were also examined to preliminarily assess their potential for release as biological control agents (Holthouse et al. 2020, Kinyanjui et al. 2021).

## Materials and Methods

### Summary of sampling sites

**Figure 1.**
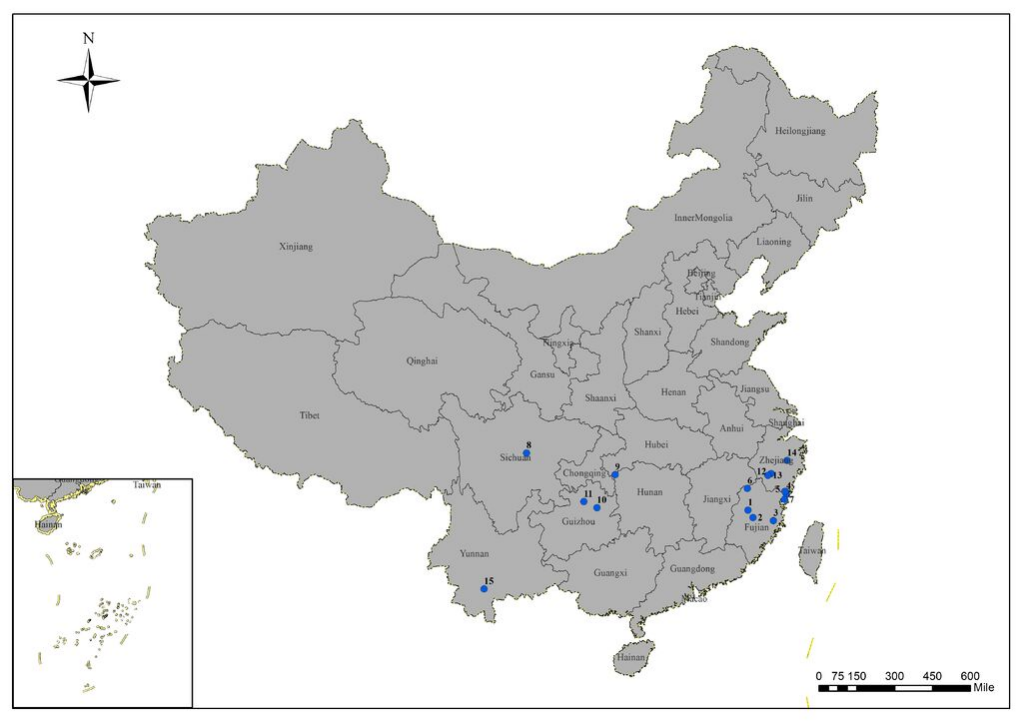
Blue dots indicate deployment and collection sites of wild larvae and pupae masses in China, 2016-2019. *Dendrolimushoui* was discovered at Sites 1 and 15. Geographical coordinates are as follows: Site 1 (FJG): 26°35'33.23" N, 117°31'17.18" E; Site 2 (FSC): 26°8'28.94" N, 117°45'31.43" E; Site 3 (FYD): 25°46'29.49" N, 118°59'8.37" E; Site 4 (FFY): 27°7'41.18" N, 120°5'5.14" E; Site 5 (FFC): 27°16'46.26" N, 120°2'13.21" E; Site 6 (FWW): 27°48'52.32" N, 117°42'36.16" E; Site 7 (FXY): 26°49'47.74" N, 119°54'28.67" E; Site 8 (SCZ): 30°45'1.26" N, 103°28'30.72" E; Site 9 (HLG): 29°24'45.20" N, 109°18'5.50" E; Site 10 (GSS): 27°34'56.05" N, 108°1'49.70" E; Site 11 (GQL): 27°56'55.15" N, 107°11'56.94" E; Site 12 (ZSN): 28°24'46.33" N, 119°22'35.09" E; Site 13 (ZSG): 28°21'21.1" N, 119°8'40.91" E; Site 14 (ZPD): 28°58'57.84" N, 120°33'4.8" E; Site 15 (YPJ) 22°59'36.13" N, 101°05'31.39" E.

According to the distribution of *C.japonica* forest and its infestation by *D.houi*, 15 representative sites were located and investigated in five provinces in China (Fig. 1): Fujian (26°35′33.23″ N, 117°31′17.18″ E, 882 m a.s.l., Guiyang Village, Jiangle County, Fujian Province, FJG; 26°8′28.94″ N, 117°45′31.43″ E, 901 m a.s.l., Chengqian Village, Sha County, Fujian Province, FSC; 25°46′29.49″ N, 118°59′8.37″ E, 915 m a.s.l., Duishan Village, Yongtai County, Fujian Province, FYD; 27°7′41.18″ N, 120°5′5.14″ E, 422 m a.s.l., Youkeng Village, Fuding County, Fujian Province, FFY; 27°16′46.26″ N, 120°2′13.21″ E, 603 m a.s.l., Chayang Village, Fuding County, Fujian Province, FFC; 27°48′52.32″ N, 117°42′36.16″ E, 1108 m a.s.l., Wuyi Mountain, Wuyishan County, Fujian Province, FWW; and 26°49′47.74″ N, 119°54′28.67″ E, 578 m a.s.l., Yangmeiling Forest Park, Xiapu County, Fujian Province, FXY), Sichuan (30°45′1.26″ N, 103°28′30.72″ E, 662 m a.s.l., Zuling Temple, Chongzhou County, Sicuan Province, SCZ), Hubei (29°24′45.20″ N, 109°18′5.50″ E, 895 m a.s.l., Guimao Mountain, Laifeng County, Hubei Province, HLG), Guizhou (27°34'56.05″ N, 108°1'49.70″ E, 787 m a.s.l., Sanxing Village, Sinan County, Guizhou Province, GSS; 27°56′55.15″ N, 107°11′56.94″ E, 1022 m a.s.l., Longli County, Qiangnan County, Guizhou Province, GQL), Yunnan (22°59′36.1 3″ N, 101°05′31.39″ E, 561 m a.s.l. Jingdong Yi Autonomous County, Pu'er City, Yunnan Province, YPJ) and Zhejiang (28°24′46.33″ N, 119°22′35.09″ E, 462 m a.s.l., Nandai Village, Songyang County, Zhejiang Province, ZSN; 28°21′21.1″ N, 119°8′40.91″ E, 1002 m a.s.l., Guiyang Village, Suichang County, Zhejiang Province, ZSG; 28°58′57.84″ N, 120°33′4.8″ E, 698 m a.s.l., Dapan Forest Farm, Pan'an County, Zhejiang Province, ZPD). Different samples of *D.houi* were individually collected, numbered and reared separately.

### Collection, rearing and identification of parasitic wasps and parasitic flies

**Figure 2.**
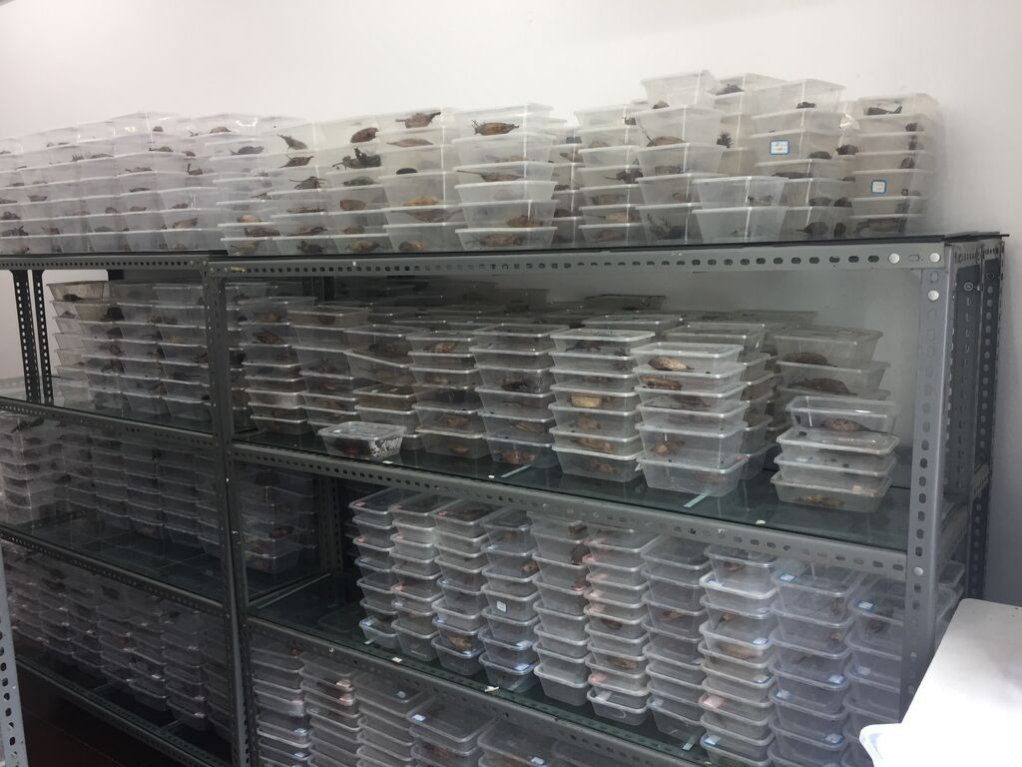
Mass rearing of *Dendrolimushoui*.

**Figure 3.**
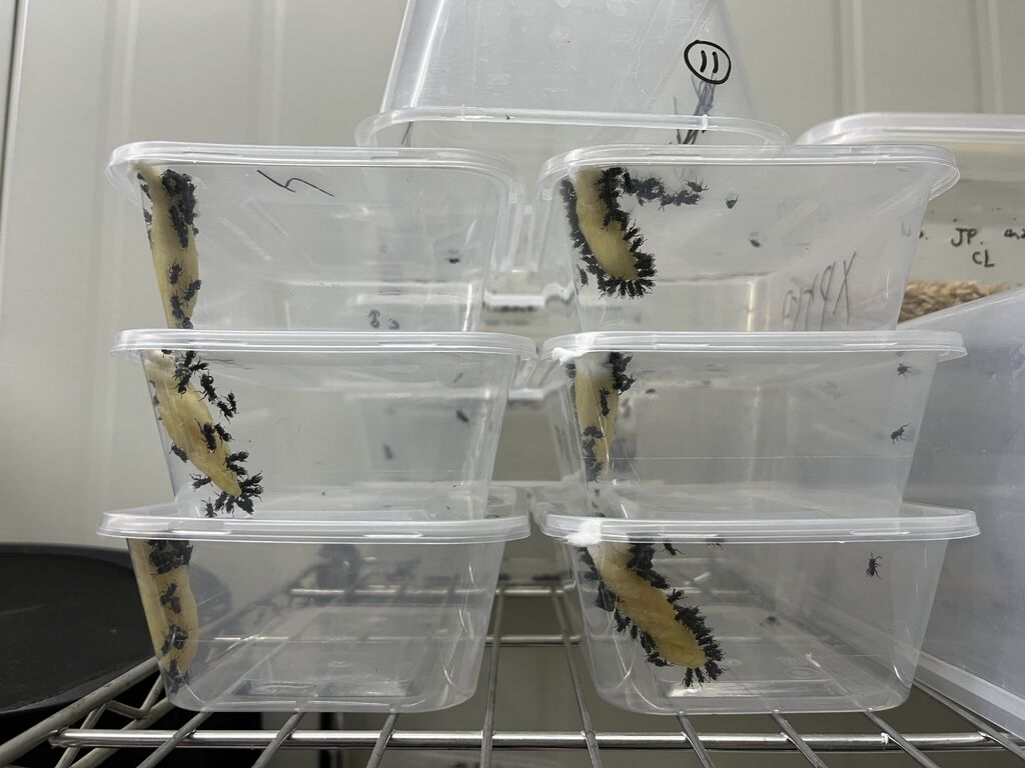
Rearing of parasitoid wasps.

In the places described, branches containing pupae of *D.houi* were removed and observed in the lab. The hosts were maintained at 26 ± 1℃, 50 ± 10% relative humidity (R.H.) and 12/12 h light/dark (L.D.) photoperiod for 25 days during which they emerged (Fig. 2). All parasitoids were collected and fed with 100% honey in a screened plastic box (29 × 17 × 9 cm) at 25℃ and 12:12 h L.D. photoperiod after emerging from the hosts (Fig. 3). Then, all the specimens were kept in 75% ethanol. The morphological characteristics of parasitoids were observed under a microscope (SZ760B, Optec, Chongqing, China). Some specimens were sent to senior taxonomists for species identification after all specimens were preliminarily identified, based on morphological characteristics according to Huang (2003a), Huang (2003b), Yang et al. (2015) and Zhang (2016). Voucher specimens were preserved at Fujian Agriculture and Forestry University.

### Biological characteristics of parasitoids and determination of dominant parasitoids

During the rearing process (26 ± 1°C, 50 ± 10% R.H.), after adults emerged, the number of offspring was recorded every day, distinguishing between male and female by ovipositor or body size. The important parasitoid species were selected from a list of parasitic wasps and parasitic flies, based on mean criteria including: parasitism rate, sex ratio, longevity and fecundity. The parasitism rate was calculated by dividing all the emerged parasitoids by all the host pupae.

Parasitoid emergence rate was calculated as the number of parasitoids emerging from the host divided by the total number of pupae multiplied by 100. The sex ratio was calculated as the number of females divided by the number of males. Longevity was the time period from emergence of parasitoids to death. Fecundity was the number of offspring per pupa.

## Results

### Some parasitoid species within D.houi pupae and other hosts

A total of 13 species from five families and two orders of parasitoids were identified: five parasitoid species from three families containing *Xanthopimplakonowi* (Krieger), *Habronyxpyretorum* (Cameron), *Theroniadepressa* (Gupta), *Kriechbaumerelladendrolimi* (Sheng et Zhong) and *Dibrachysyunnanensis* (Yang). There are eight parasitoid flies from five genera and two families containing Carcelia (Carcelia) illiberisi Chao et Liang, Carcelia (Carcelia) nigrantennata Chao et Liang, Carcelia (Carcelia) flavimaculata Sun et Chao, Drino (Palexorista) inconspicuoides (Baranov), *Blepharipazebina* (Walker), *Mikiatepens* (Walker), Sarcophaga (Sarcorohdendorfia) gracilior (Chen) and *Sarrorohdeneantelope* (Bottcher), of which, *H.pyretorum*, *C.flavimaculata* and *M.tepens* were new parasitoids recorded for *D.houi* (Table 1).

### Regional distribution, parasitism and host stage of parasitoids within D.houi

A total of 4537 pupae of *D.houi* were collected in the field and 417 pupae were parasitised. Five parasitoid species emerged in *D.houi* pupal stage, these being *X.konowi*, *H.pyretorum*, *T.depressa*, *K.dendrolimi* and *D.yunnanensis* (Table 2) and eight parasitic flies: *C.illiberisi*, *C.nigrantennata*, *C.flavimaculata*, *D.inconspicuoides*, *M.tepens*, *B.zebina*, *S.gracilior* and *S.antelope* (Table 3). We currently found that *H.pyretorum* and *S.antelope* were newly reported in Fujian Province and *K.dendrolimi* was newly reported in Guizhou Province, China.

### Biology of dominant species

*K.dendrolimi* had parasitism rates of 3.60% during the pupal stage with various elevation distributions (462-901 m). The sex ratio (female : male) was 1 : 0.25, it had the female and male longevity within 11 d and 10 d, respectively (Table 4). Further findings showed that multiparasitism frequently and naturally occurs between *K.dendrolimi* and *T.depressa* and between *K.dendrolimi* and *D.yunnanensis*. Consequently, *K.dendrolimi* was identified as the important pupal parasitoid species, based on its parasitism rates (0.79-11.59%) in Hymenoptera species and offspring (26–58/pupa), which might lead to a synergistic effect on the suppression of the large pupae of *D.houi* and be a promising species for mass release to suppress caterpillars in *C.japonica* forests.

### Morphology of immature stages

**Figure 4.**
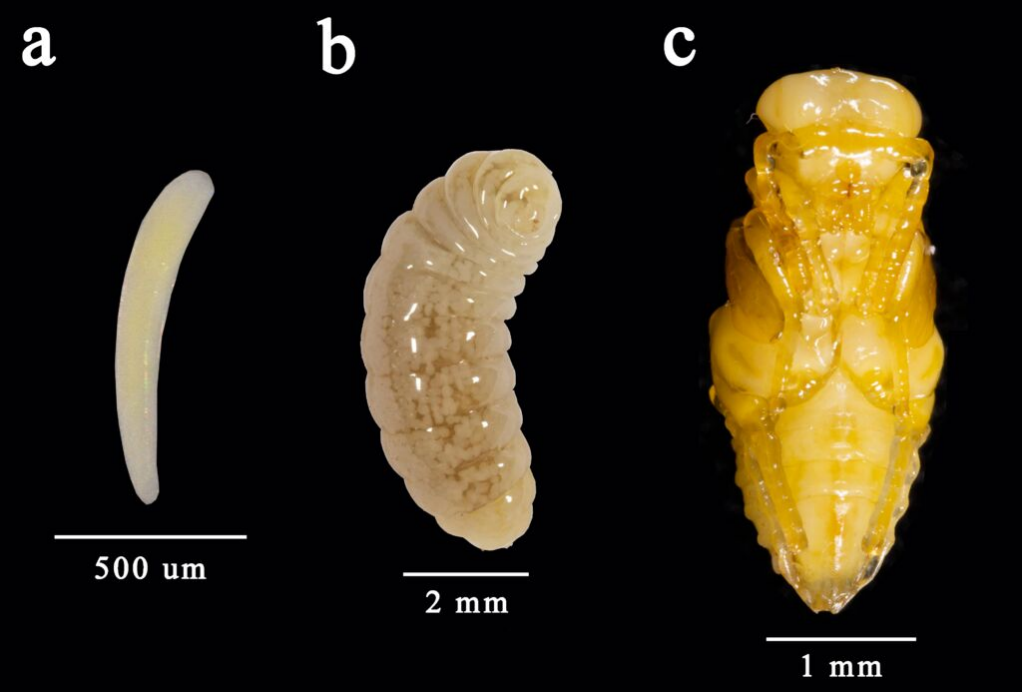
*K.dendrolimi*. **a** egg; **b** late-instar larva ; **c** pupa.

Egg clavate, light yellow, length 0.8-1.0 mm, one end is round and the other is thin and transparent. Early-instar larvae length is 1.8-5.0 mm, tawny, the whole body is covered with a transparent membrane, appears like a spindle with pointed ends. Late-instar larvae length is 6.1-8.5 mm, the body is yellowish-white, blunt round head and the tail is thin. Free pupa, with 5.5-8.0 mm length. Initial pupa stages with white, later brown (Fig. 4) and black before emergence.

### Morphology of adults

**Figure 5.**
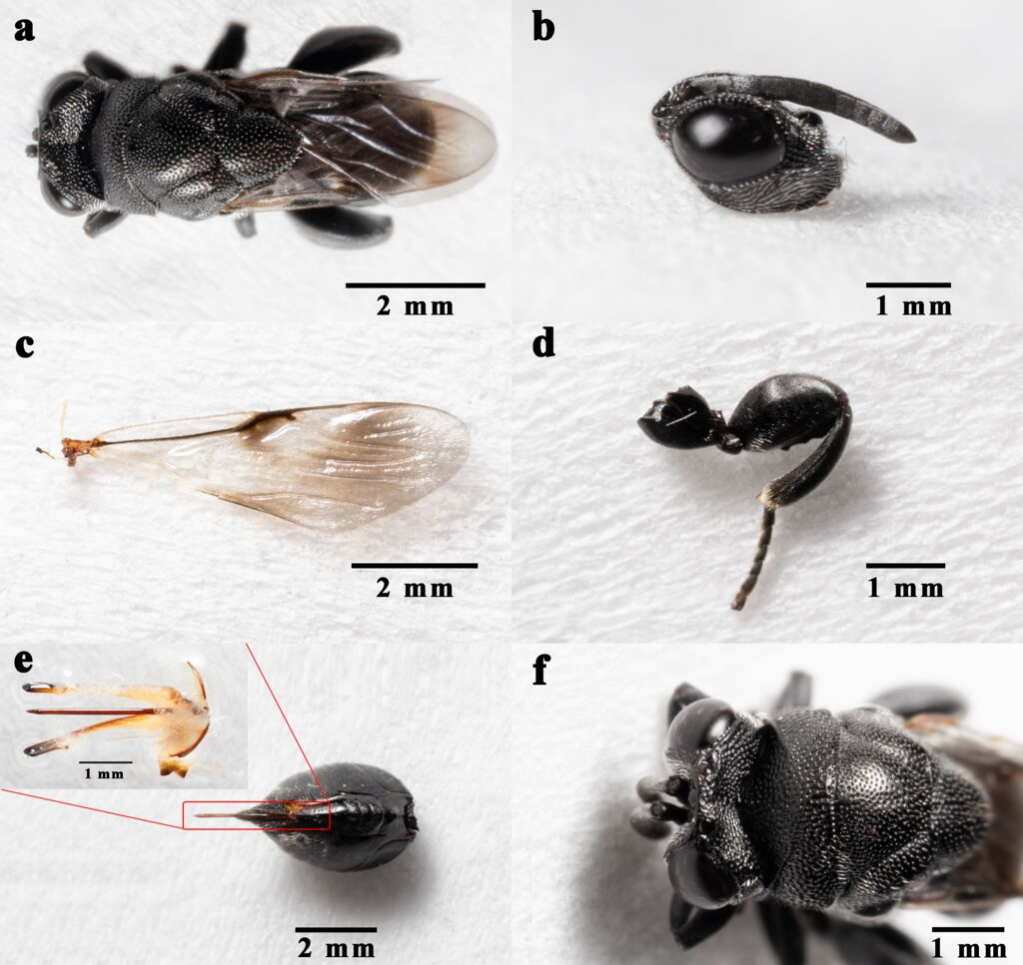
*K.dendrolimi*, female. **a** whole body; **b** head, **c** forewing; **d** hind leg, **e** cercus and ovipositor; **f** head and mesosoma in dorsal view.

The length of females is 6.8-7.2 mm, the length of males is 4.2-5.7 mm. Antennae almost blackish, scapus and anelli kermesinus, tegula dark brown, forewings faintly smoky with brown patch, hind tarsus dark brown, scapus almost reaching middle ocelli, the length is equal to the 1-4 funiculur segments. OOL : POL = 9 : 28. Blunt triangle occiput, pits on pronotum were narrower than on mesoscutum and scutellum, frenal length greater than width, back end sleek without concave edge, forewings submarginal vein 1.5 times as long as marginal, stigmal vein stubby, coxa-3 dorsal lateral base with tuberculate protuberances, hind femur length about 1.5 times the width, first gastral tergite about 2/5 the length of the cercus. The ovipositor 2 mm in length (Fig. 5).

## Discussion

In previous research, 22 different native parasitoid species from 16 genera and 11 families within *D.houi* were identified, containing seven parasitoid species that emerged from eggs: *Mesopolobustabatae* Ishii, *M.albitarsis*, *A.gastropachae*, *Ooencyrtuskuwanae* Howard, *Telenomusdendrolimi* Matsumura, *T.dendrolimusi* Chu and *M.subfumatus* (Ratzeburg); nine species of parasitoid species emerged from the pupae of *D.houi*, including *K.dendrolimi*, *K.longiscutellaris* Qian and He, *BrachymeriaSecundaria* Rushika, *B.lasus*, *Monodontomerusminor* Ratz, *D.yunnanensis*, *T.depressa*, *Coccygomimuslaothoe* Cameron and *X.konowi*. Here, we identified five species of parasitoid species, of which a new parasitoid species was discovered and identified compared with previous studies: *Habronyxpyretorum* (Lin et al. 2017). Seven different native parasitoid flies from four genera and two families within *D.houi* were identified, these being *C.rasella*, *C.illiberisi*, *C.nigrantennata*, *D.inconspicuoides*, *B.zebina*, *S.gracilior* and *S.antilope*. They parasitised *D.houi* larvae and emerged in its pupal stage. We identified eight species of parasitoid flies from five genera and two families, of which *Carceliaflavimaculata* and *Mikiatepens* were new parasitoid flies recorded for *D.houi* (Liang et al. 2018).

There are four species of parasitoid natural enemies distributed only in China, *Habronyxpyretorum*, *Kriechbaumerelladendrolimi*, *Dibrachysyunnanensis* and *Carcelianigrantennata* mainly distributed in south China. *Carceliailliberisi* and *Carceliaflavimaculata* have a naturally broad distribution in a large latitude crossing (Tong and Ni 1986, Zhao and Liang 2002, Huang 2003a, Huang 2003b). The following seven species of parasitic wasps and parasitoid flies are found in other countries besides China. *Xanthopimplakonowi* is distributed in Japan, India, Myanmar, Vietnam, Thailand, Malaysia and Indonesia (Huang 2003a). *Theroniadepressa* has been recorded in south China as well as in the Philippines (Lin et al. 2017). *Drinoinconspicuoides* is distributed in Japan and Melanesia; *Blepharipazebina* in addition to 29 provinces in China, with distribution in Russia, South Korea, India, Nepal, Myanmar, Thailand and Sri Lanka. *Mikiatepens* was a commonly distributed species in Russia, Japan, Kazakhstan, India, Bhutan, Nepal, Bangladesh, Vietnam and Malaysia (Zhang 2016). *Sarcophagagracilior* was distributed in south China and Nepal, while *Sarrorohdeneantelope* is distributed abroad in Russia, Korea, Japan and northern Oceania (Fan 1992).

Further findings showed that multiparasitism frequently and naturally occurs between *K.dendrolimi* and *T.depressa*, between *K.dendrolimi* and *D.yunnanensis* and between parasitoid flies *Carcelianigrantennata*, *Blepharipazebina* and *Sarcophagagracilior*. The results showed that multiple natural enemies could attack the same large host at the same time, which might lead to a synergistic effect on the suppression of the large pupae of *D.houi* and be a promising species for mass release to suppress caterpillars in *C.japonica* forests. Obviously, multiple natural enemies co-exist in the pupae of *D.houi*, killing host pests through feeding, which has a combined control effect on the natural population. However, this phenomenon is contrary to the law of species competition, so it is considered as a strange and interesting phenomenon (Hackett-Jones et al. 2008). Due to the large size gap between natural enemies and hosts, a single parasitoid species cannot overcome the defence of hosts' immune systems and successfully inhibit host development and multiple natural enemies are required to participate at the same time. Therefore, polyparasitism or multiparasitism may have more potential for pest control (Yang et al. 2018). It is worthy of further study to provide reference for the protection and rational utilisation of natural enemies in the future.

Previous studies revealed that *K.dendrolimi* was originally recorded attacking large moths, such as *D.punctatus*, *D.kikuchii* and *D.houi*, while *A.pernyi*, *Philosamiacynthia* Walker and Felder, *Lebedanobilis* Walker, *E.pyretorum* and *Trabalavishnou* Lefebure are factitious hosts (Tong and Ni 1986). Unfortunately, little was known about the interaction between these host pests and parasitoid *K.dendrolimi*, including the mass rearing procedure (Suppl. materials 1, 2). Amongst the host pupae in this paper, *A.pernyi* might be associated with being larger and heavier and having more abundant nutrients within one pupa (King 1987), which is sufficient to simultaneously support hundreds of parasitoid larvae inside them to develop into adults. Additionally, *A.pernyi* pupae are easily and extensively accessible from the north of China at low cost for mass rearing of *K.dendrolimi*. A high ratio of female parasitic wasps may increase the outcome of offspring in the next generation, which is beneficial to the expansion of parasitoid population (Uckan and Gulel 2002).

The average annual temperature of *K.dendrolimi* collected in Guizhou Province was 15.3°C; therefore, *K.dendrolimi* was able to adapt to lower temperatures. The laboratory rearing data could predict its potential distribution and evaluate its pest control efficiency in the forest. In addition, *K.dendrolimi* were better reared under 24°C-30°C, which could be used to optimise and regulate rearing conditions and change their development process according to practical need. All results will be conducive to mass rearing and release in the future (Pereira et al. 2011, Bao et al. 2021).

As a native parasitoid species, *K.dendrolimi* has stronger adaptability to local stress factors than imported species and it has a high emergence rate from wild populations of *D.houi* with strong fertility and it specially attacks most Lasiocampidae pests. Therefore, this parasitoid species will be a potentially promising biological control agent for control of pine caterpillars in China. Using natural enemies to control *D.houi* has some advantages, such as stronger specificity, better environmental protection (Gurr et al. 2011) and *K.dendrolimi* can be industrialised to become an economic control agent by providing sufficient supply of alternative hosts. For releasing purpose, the population needs to be increased by mass rearing prior to the outbreak of *D.houi* and transported artificially before jump-like releasing or inundant releasing in the field (Rossi Stacconi et al. 2018).

## Supplementary Material

AFBA9CA9-AADA-5777-88E4-6876F565E958 10.3897/BDJ.11.e97878.suppl1 Supplementary material 1 The courtship behaviour of *Kriechbaumerelladendrolimi* Data type multimedia File: oo_822946.mp4 https://binary.pensoft.net/file/822946 ZhengHao Chen

49859EA8-CCB1-55F7-8295-6354F5F4C0FA 10.3897/BDJ.11.e97878.suppl2 Supplementary material 2 Mating behaviour of Kriechbaumerelladendrolimi Data type multimedia File: oo_823085.mp4 https://binary.pensoft.net/file/823085 Zheng-Hao Chen

## Figures and Tables

**Table 1.** Parasitc species within *D.houi* pupae and other hosts.

NO.	Order	Family	Species	Hosts	References
1	Hymenoptera	Ichneumonidae	*Xanthopimplakonowi*	*D.houi*, *D.punctatus*, *Attacusarlas*, *Antheraeapernyi*, *Philosamiacynthia*, *Saturniapyretorm*, *Antheraeafrithi*, *Antheraeapolyphemus*, *Attacusdohertyi*, *Criculatrifenestrata*, *Malacosomaneustriatestacea*	Huang (2003a)
2			*Habronyxpyretorum*	*D.houi, *Dictyoplocajaponica*, *E.pyretorum*	Huang (2003a)
3			*Theroniadepressa*	*D.houi*, *Artonafuneralis*	Huang (2003a)
4		Chalcididae	*Kriechbaumerelladendrolimi*	*D.houi*, *D.kikuchii*, *D.punctatus*	Tong and Ni (1986)
5		Pteromalidae	*Dibrachysyunnanensis*	*D.houi*, *Tomicuspiniper*	Jiao et al. (2017)
6	Diptera	Tachinidae	*Carceliailliberisi*	*D.houi*, *Illiberispruni*	Zhao and Liang (2002)
7			*Carcelianigrantennata*	*D.houi*, *Lymantriadispar*, *Euproctissimiles*	Huang (2003b)
8			*Carceliaflavimaculata*	*D.houi, *Lymantriaxylina*, *Diprionjingyuanensis*	Zhao and Liang (2002)
9			*Drinoinconspicuoides*	*D.houi*, *L.xylina*	Zhang (2016)
10			*Blepharipazebina*	*D.houi*, *D.punctata*, *D.kikuchii*, *Dendrolimussuperans*, *Antheraeamylitta*, *Cephonodeshylas*, *Papiliodemoleus*, *Andracabipunctata*, *Hepialusyunnanensis*, *Ivelaochropoda*, *Dasychiraaxutha*	Zhang (2016)
11			*Mikiatepens*	*D.houi	Zhang (2016)
12		Sarcophagidae	*Sarrorohdenegracilior*	*D.houi*	Fan (1992)
13			*Sarrorohdeneantelope*	*D.houi*	Fan (1992)

* New parasitoid wasps or parasitoid flies recorded within *Dendrolimushoui*.

**Table 2.** Percent parasitism and distribution of parasitoid wasps within pupae of *D.houi* in China. (Guiyang Village, Jiangle County, Fujian Province, FJG; Chengqian Village, Sha County, Fujian Province, FSC; Duishan Village, Yongtai County, Fujian Province, FYD; Youkeng Village, Fuding County, Fujian Province, FFY; Chayang Village, Fuding County, Fujian Province, FFC; Wuyi Mountain, Wuyishan County, Fujian Province, FWW; Yangmeiling Forest Park, Xiapu County, Fujian Province, FXY; Zuling Temple, Chongzhou County, Sicuan Province, SCZ; Guimao Mountain, Laifeng County, Hubei Province, HLG; Sanxing Village, Sinan County, Guizhou Province, GSS; Nandai Village, Songyang County, Zhejiang Province, ZSN; Guiyang Village, Suichang County, Zhejiang Province, ZSG; Dapan Forest Farm, Pan'an County, Zhejiang Province, ZPD)

		Parasitism rate (%) in different localities during the pupae stage												
Species	Offspring	FJG	FSC	FYD	FFY	FFC	FXY	FWW	SCZ	HLG	GSS	ZSN	ZSG	ZPD
*X.konowi*	1	0.52			0.22	0.36	0.16							
*H.pyretorum*	1			0.48		0.36	0.79	4.35	2.44	0.62		0.98	4.17	0.47
*T.depressa*	1-9	0.86	0.48			0.36	0.63	4.35						
*K.dendrolimi*	26-58	4.30	1.39				0.32	11.59			0.79	3.92		5.14
*D.yunnanensis*	33-49			0.48										

**Table 3.** Percent parasitism and distribution of parasitic flies within pupae of *D.houi* in China. (Guiyang Village, Jiangle County, Fujian Province, FJG; Chengqian Village, Sha County, Fujian Province, FSC; Duishan Village, Yongtai County, Fujian Province, FYD; Yangmeiling Forest Park, Xiapu County, Fujian Province, FXY; Zuling Temple, Chongzhou County, Sicuan Province, SCZ; Guimao Mountain, Laifeng County, Hubei Province, HLG; Longli County, Qiangnan County, Guizhou Province, GQL; Jingdong Yi Autonomous County, Pu'er City, Yunnan Province, YPJ; Dapan Forest Farm, Pan'an County, Zhejiang Province, ZPD)

Species	Offspring	FJG	FSC	FYD	FXY	SCZ	HLG	GQL	YPS	ZPD
*C.illiberisi*	1-3			0.32						
*C.nigrantennata*	1-5	3.79	1.39	1.29		2.44	1.85			5.78
*C.flavimaculata*	1-5									0.32
*D.inconspicuoides*	1		0.55							
*B.zebina*	1-11	3.79	5.54	2.89				3.33		
*M.tepens*	1								1.54	
*S.gracilior*	4-8	1.89	8.31	3.38	4.27		0.31			3.69
*S.antelope*	3-8	2.41								

**Table 4.** Biology of high parasitism rates parasitoid species.

Species	Total parasitism rates (%)	Order	A.S.L. (m)	Offspring	Ratio female : male	Longevity (d)
*K.dendrolimi*	3.19	Hymenoptera	462-901	26-58	1 ：0.25	8-16
*S.gracilior*	3.59	Diptera	578-915	1~14	1 ：0.67	8-12
